# In-vivo optical detection of cancer using chlorin e6 – polyvinylpyrrolidone induced fluorescence imaging and spectroscopy

**DOI:** 10.1186/1471-2342-9-1

**Published:** 2009-01-08

**Authors:** William WL Chin, Patricia SP Thong, Ramaswamy Bhuvaneswari, Khee Chee Soo, Paul WS Heng, Malini Olivo

**Affiliations:** 1 Division of Medical Sciences, National Cancer Centre Singapore, 11 Hospital Drive, 169610, Singapore; 2 Department of Pharmacy, National University of Singapore, Block S4, 18 Science Drive 4, Singapore, 117543; 3 Singapore Bioimaging Consortium, Biomedical Sciences Institutes, 11 Biopolis Way, #02-02 Helios, 138667, Singapore

## Abstract

**Background:**

Photosensitizer based fluorescence imaging and spectroscopy is fast becoming a promising approach for cancer detection. The purpose of this study was to examine the use of the photosensitizer chlorin e6 (Ce6) formulated in polyvinylpyrrolidone (PVP) as a potential exogenous fluorophore for fluorescence imaging and spectroscopic detection of human cancer tissue xenografted in preclinical models as well as in a patient.

**Methods:**

Fluorescence imaging was performed on MGH human bladder tumor xenografted on both the chick chorioallantoic membrane (CAM) and the murine model using a fluorescence endoscopy imaging system. In addition, fiber optic based fluorescence spectroscopy was performed on tumors and various normal organs in the same mice to validate the macroscopic images. In one patient, fluorescence imaging was performed on angiosarcoma lesions and normal skin in conjunction with fluorescence spectroscopy to validate Ce6-PVP induced fluorescence visual assessment of the lesions.

**Results:**

Margins of tumor xenografts in the CAM model were clearly outlined under fluorescence imaging. Ce6-PVP-induced fluorescence imaging yielded a specificity of 83% on the CAM model. In mice, fluorescence intensity of Ce6-PVP was higher in bladder tumor compared to adjacent muscle and normal bladder. Clinical results confirmed that fluorescence imaging clearly captured the fluorescence of Ce6-PVP in angiosarcoma lesions and good correlation was found between fluorescence imaging and spectral measurement in the patient.

**Conclusion:**

Combination of Ce6-PVP induced fluorescence imaging and spectroscopy could allow for optical detection and discrimination between cancer and the surrounding normal tissues. Ce6-PVP seems to be a promising fluorophore for fluorescence diagnosis of cancer.

## Background

As with most cancers, early diagnosis is critical to achieve favorable prognosis. Currently, random surveillance biopsies are the existing gold standard for the identification of lesions in pre-neoplastic conditions. However this method is prone to sampling error, time-consuming, subjective and cost-inefficient. A diagnostic method that could provide rapid, automated classification of cancer lesions would increase the efficiency and comprehensiveness of malignancy screening and surveillance procedures. A variety of optical techniques have recently been utilized for the diagnostic study of cancerous tissue. These include fluorescence spectroscopy [1], Raman spectroscopy [2], light scattering spectroscopy [3], and Fourier-transform infrared spectroscopy [4]. These optical spectroscopic techniques are capable of providing biochemical and morphological information in short integration times, which can be used for automated diagnosis of intact tissue. However, in order to be useful as a comprehensive screening procedure, the optical technique must allow rapid real time imaging of a large area of tissue rather than point by point measurement, such that suspicious regions could be identified accurately and biopsied for histopathologic correlation [5].

With the advent of molecular probes, imaging methods such as ultrasound, microCT (Computed Tomography), microMRI (Magnetic Resonance Imaging), and microPET (Positron Emission Tomography) can be conducted not only to visualize gross anatomical structures, but also to visualize substructures of cells and monitor molecule dynamics [6]. Imaging of endogenous or exogenous fluorochromes has several important advantages over other optical approaches for tumor imaging. This imaging technique relies on fluorochrome induced fluorescence, reflectance, absorption or bioluminescence as the source of contrast, while imaging systems can be based on diffuse optical tomography, surface-weighted imaging, phase-array detection, intensified matrix detector and charged-coupled device camera detection, confocal endomicroscopy, multiphoton imaging, or microscopic imaging with intravital microscopy [7,8]. Fluorescence ratio imaging is a method widely used for optical diagnosis of cancer after administration of a photosensitizer [9]. Enhanced contrast between tumor and adjacent normal tissue can be obtained based on calculating the ratio between red intensity of the photosensitizer (600–700 nm) over the blue/green intensity of the back-scattered excitation light or tissue autofluorescence (450–550 nm). Many investigations have confirmed good agreement with the histopathological extent of the tumor, implying that this technique can be applied as a useful tool for indicating tumor boundary [10].

A number of fluorochromes such as fluorescein, toluidine blue, cyanine dyes and indocyanine green have been described with variable stabilities, quantum efficiencies, and ease of synthesis. However, most of the fluorochromes are not tumor specific and are rapidly eliminated from the organism. Chemically and endogenously synthesized fluorochromes such as porphyrin based photosensitizers have properties that may be utilized both experimentally and clinically. Porphyrins have been known to naturally localize in malignant tissue where they emit light when irradiated at certain wavelengths, providing a means to detect tumor by the location of its fluorescence. However, one of the major limitation is its slow clearance from tissues and long period of skin phototoxicity. Moreover, the porphyrin's core absorbs wavelengths of light too short for optimal penetration in tissue. As such, by reducing a pyrrole double bond on the porphyrin periphery, a chlorin core compound can be generated with a high absorption at longer wavelengths of 660 – 670 nm that can penetrate deeper in human tissue than those of porphyrins. Of particular interest among the evaluated chlorins is the naturally occurring chlorin e6 (Ce6) [11]. Ce6 has improved efficacy and has decreased side effects compared to first generation photosensitizers from hematoporphyrin derivatives. Due to the importance of Ce6's characteristic fluorescence properties, there is a need to identify new formulations that are stable, exhibit ease in manufacturing and selectively deliver the photosensitizer to target tissue in an efficient manner. Hence, we have investigated the use of Ce6 in combination with the polymer polyvinylpyrrolidone (Ce6-PVP). Polyvinylpyrrolidone is one of the most important excipient used in modern pharmaceutical technology. We have previously described the selective localization and photodynamic activity of Ce6-PVP in nasopharyngeal and lung carcinoma models that provided rationale for its use as a therapeutic agent for photodynamic therapy [12,13]. By employing a chick chorioallantoic membrane model, Ce6-PVP was shown to selective accumulate in bladder tumors xenografts and had a faster clearance from normal CAM when administered topically compared to systematic administration [14]. The uptake ratio of Ce6-PVP was found to have a 2-fold increase across the CAM when compared to that of Ce6, indicating that PVP was able to facilitate diffusion of Ce6 across the membrane [15]. Furthermore, Ce6-PVP had less in vivo systemic phototoxic effect compared to Ce6 alone after light irradiation in photodynamic therapy in mice bearing tumors [16]. Using a chemical fluorescence extraction technique and cuvette-based spectrofluorimetry, our data demonstrated that the distribution of Ce6-PVP drug were much lower in normal organs such liver, spleen, kidney, brain, heart and lung compared to Ce6 delivered using dimethylsulfoxide (DMSO) [17]. We also postulated that the extent of tumor necrosis post Ce6-PVP mediated photodynamic therapy (PDT) was dependent on the plasma concentration of Ce6-PVP, implying a vascular mediated cell death mechanism [18].

In this study, we have evaluated the usefulness of Ce6-PVP to accurately define the margin of the tumor from its normal adjacent tissue in the chick choriallantoic membrane (CAM) tumor model. We also presented visual information on Ce6-PVP induced fluorescence in tumor and gross anatomical structure of normal organ of a murine model. Fluorescence spectroscopy measurements were also performed to characterize emission spectra from these tissue samples as well as to corroborate results from fluorescence images. Finally, a pilot trial was carried out to validate the use of Ce6-PVP as a clinically relevant diagnostic photosensitizer using both imaging and spectroscopy modality for differentiation of normal and tumor tissue in a patient.

## Methods

### Photosensitizer

The formulation of Ce6-PVP, also known as Fotolon or Photolon was supplied by HAEMATO-science GmbH, Germany. It is a co-lyophilisate of Ce6 sodium salt and PVP (a pharmaceutical grade polymer, molecular mass ≈ 12,000 g/mol) in a 1:1 mass ratio [19].

### Cell culture

MGH (European Collection of Cell Cultures), a poorly differentiated human bladder carcinoma cells were grown as a monolayer in RPMI-1640 medium supplemented with 10% fetal bovine serum, 1% non-essential amino acids (Gibco, USA), 1% sodium pyruvate (Gibco, USA), 100 units mL^-1 ^penicillin-streptomycin (Gibco, USA) and incubated at 37°C, 95% humidity and 5% CO_2_.

### Chick choriallantoic membrane tumor model

Fertilized chicken eggs were incubated at 37°C in a humidified atmosphere inside a hatching incubator equipped with an automatic rotator (Octagon 20, Brinsea, Somerset, UK). At embryo age (EA) 7, a window of about 1.5 cm was opened in the eggshell to detach the shell membrane from the developing CAM. Then, the window was sealed with sterilized parafilm to avoid contamination and the eggs were returned to the static incubator for further incubation until the day of experiment. On EA 9, approximately 5–10 × 10^6 ^MGH cells were inoculated on the CAM. The window of the eggs were resealed with sterile parafilm and returned to the static incubator. Grafted cells were allowed to grow on the CAM for up to 5 days. On EA 14, Ce6-PVP was dissolved in 0.9% sodium chloride to constitute a stock solution of 1 mg/mL. The stock solution was further diluted to obtain a volume of 500 μL containing a dose of 1 mg/kg body weight of the chick's embryo. The photosensitizer was applied on the entire surface of the CAM and left to incubate for 30 min. The window was resealed to avoid evaporation of the drug solution from the CAM. After 30 min incubation, macroscopic fluorescence imaging was performed at 0.5, 1, 2, 3, 4, and 5 h post drug administration using a commercially available fluorescence endoscopic system (Karl Storz, Tuttlingen, Germany). A modified xenon short arc lamp (D – Light system in blue light mode, Karl Storz) filtered by a band pass filter (380 – 450 nm) was used for excitation of photosensitizer in tissue. Fluorescence was captured via a sensitive CCD camera (Tricam SL PAL, Karl Storz) attached to an endoscope integrated with a long pass filter (cut-off wavelength 470 nm). This observation LP filter of the endoscope only minimally transmits the diffuse back-scattering excitation light with a peak at 450 nm (blue light), while has a transmission of over 98% in the 470 – 800 nm range. The red channel registered the photosensitizer's fluorescence and the blue channel captured the diffusely back-scattered excitation light. A short exposure of the surface of tissue to the excitation light (10 s) was performed to avoid excessive photobleaching effects. White light imaging was used to correlate the boundaries of tumors and organs. All procedures involving preparation and administration of the photosensitizer were conducted under low ambient lighting.

### Murine tumor model

A total of 10 Balb/c athymic nude mice and C57 mice, 6–8 weeks of age, weighing an average of 24 g were obtained from the Animal Resource Centre, Western Australia and Centre for Animal Resources, National University of Singapore respectively. Before inoculation, the cell layer was washed with phosphate-buffered saline, trypsinized, and counted using a hemocytometer. Approximately 3.0 × 10^6 ^MGH cells suspended in 150 μl of Hanks' Balanced Salt Solution (Gibco, USA) were injected subcutaneously into the lower flanks of Balb/c athymic nude mice. The animals were used for experiments when the tumors measured around 7 – 10 mm in diameter. This ensured that the tumor sizes were kept consistent to minimize variations due to the degree of vascularization of the implants. Mice were injected with a dose of 5 mg/kg of Ce6-PVP via tail vein injection. At 1, 3 and 6 h, mice were sacrificed and the skin overlaying the tumor was carefully removed to expose the tumor and normal peritumoral muscle for fluorescence imaging. C57 mice were used for imaging and spectroscopy of normal organs. All procedures were approved by the Institutional Animal Care and Use Committee, SingHealth, Singapore, in accordance with international standards.

### Spectroscopic measurement using fiber optics-based fluorescence spectrometer

The spectral measurement was performed on mice sacrificed at 1 and 3 h post Ce6-PVP administration. A fiber optics-based fluorescence spectrometer (Spex SkinSkan, JY Inc., Edison, NJ, USA) was used for the measurement of fluorescence intensity of Ce6-PVP. A monochromator with a 150-W Xenon lamp was used as the excitation light source. The excitation light (400 nm) was guided to illuminate samples by one arm of a Y-type quartz fiber bundle, and the emission fluorescence was collected by another arm of the fiber bundle, guided to another motor-controlled monochromator. The resulting emission spectra were recorded from 650 to 750 nm, in 1 nm increments, collected using the DataMax version 2.20 (Instruments SA, Inc.) software package. The optical fiber tip was placed on the measuring sites and fluorescence intensity spectra were measured. After each measurement, the optical fiber tip was carefully cleaned to remove the possible remaining drug on the tip.

### Human subject

After informed consent, 1 patient with histologically proven angiosarcoma was recruited in this pilot case study. One tumor was located on the scalp, and 2 at the temperomandibular joint. These tumors had been previously treated with Ce6-PVP photodynamic therapy. The patient was intravenously administered with Ce6-PVP with a dose of 2.0 mg/kg for repeated photodynamic therapy on existing and a new angiosarcoma lesion on the scalp and face. Before light irradiation, fluorescence imaging and spectroscopy were performed on 3 angiosarcoma lesions, normal scalp and skin at 1 and 3 h post drug administration. The patient had to remain in subdued light throughout the imaging period. This study was approved by the National Cancer Centre Singapore's Institutional Review Board.

### Data analysis

To evaluate the quality of discrimination between healthy and tumor tissues of the fluorescence images in the CAM model, the red to blue ratio algorithm was applied. Such algorithm is independent of the geometries of excitation/collection of signals and the power of excitation during the fluorescence imaging process. The sensitivity and the specificity of the classifier were calculated using the receiver-operator characteristics (ROC) curves by plotting the fluorescence intensity of tumor against the fluorescence intensity of normal CAM tissue using the GraphPad software (GraphPad Prism™ Version 4.03, San Diego, USA). The ROC curve illustrates the trade-off between sensitivity and specificity for the different threshold of red to blue ratios to distinguish healthy from tumor tissue. Next, the cut-off value corresponding to the highest combined sensitivity and specificity was chosen and evaluated. For fluorescence spectroscopy, the emission spectra data presented in the results are the absolute fluorescence intensities of the tissues after pre-processed using Fourier transformation to decrease noise levels and normalizing the spectra to baseline at 700 nm. The normalization procedure significantly reduced the within-class variances. Spectral data from the various organs and tissues were analyzed to determine spectral line shape and the peak fluorescence intensities at region of 660 – 690 nm.

## Results

### Fluorescence imaging of bladder tumor xenografts on the CAM model

**Figure 1:**
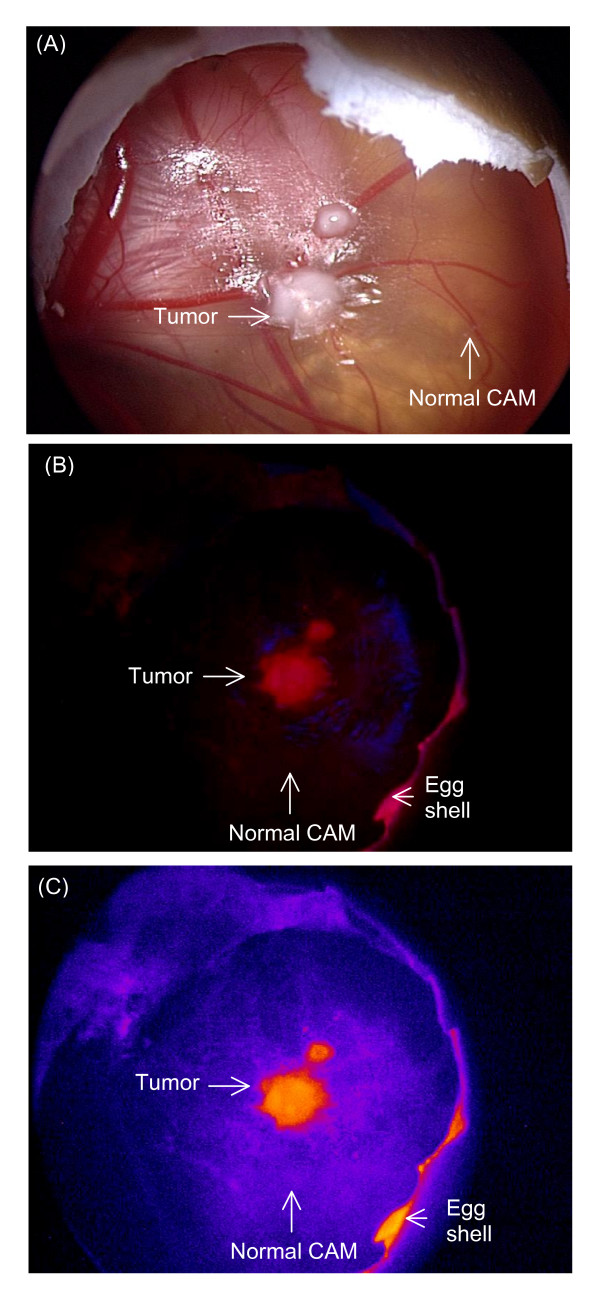
**Fluorescence imaging of MGH human bladder tumor xenografted on the CAM model**. (A) White light image of the tumor before drug administration, (B) Ce6-PVP induced red fluorescence in tumor imaged under blue light illumination at 3 h post drug administration. Minimal fluorescence was observed in the adjacent normal CAM. (C) By displaying the fluorescent image in a pseudo color using simple image processing technique, a clear discrimination of the tumor border can be visualized.

**Figure 2:**
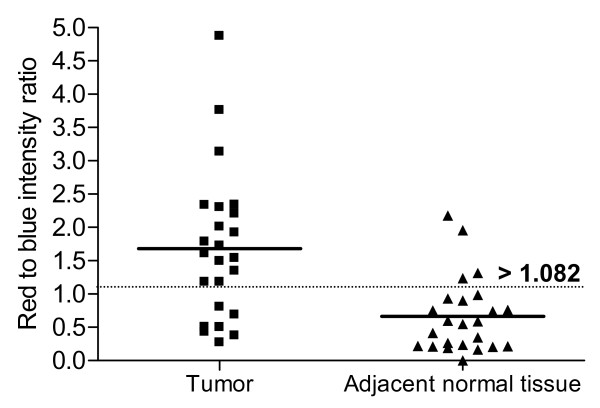
**A scatter plot comparing the fluorescence intensity in tumor and the adjacent normal CAM tissue was compiled from 1 – 5 h post topical drug administration**. The points on the scatter plot are normalized individual measurements from 24 eggs. The dotted line is the cut-off fluorescence intensity threshold derived from the ROC curve to classify tumor from normal tissue with a sensitivity and specificity of 70.8% (95% CI 48.9% to 87.4%) and 83.3% (95% CI 62.6% to 95.3%) respectively.

Fluorescence was not observed from the tumors under blue light illumination before drug administration. After topical administration of Ce6-PVP, an intense red fluorescence in the bladder tumor xenografts was observed, suggesting selective localization in the malignant cells. Fluorescence in the normal CAM tissue was lower compared to fluorescence in the tumor tissue, suggesting either a lower uptake or faster clearance rate from normal tissue of the CAM (Figure 1). The fluorescence retention from 1 to 5 h post topical administration of Ce6-PVP in bladder tumor xenografts on CAM was tabulated using the red to blue ratio algorithm and fitted into a ROC curve to validate the ability of Ce6-PVP to discriminate tumor from adjacent normal CAM membrane. By applying a cut-off value to these ratios as a diagnostic criterion, it allows the generation of sensitivity and specificity values to distinguish tumor from healthy CAM. A cut-off red to blue ratio of > 1.08 gives the highest combined sensitivity and specificity were 70.8% (95% CI 48.9% to 87.4%) and 83.3% (95% CI 62.6% to 95.3%) respectively (Figure 2). Raising the value to > 1.33 gives the sensitivity and specificity values of 62.5% (95% CI 40.59% to 81.20%) and 91.2% (95% CI 73.0% to 99.0%) respectively.

### Fluorescence imaging of tumor and normal organs in mice models

**Figure 3:**
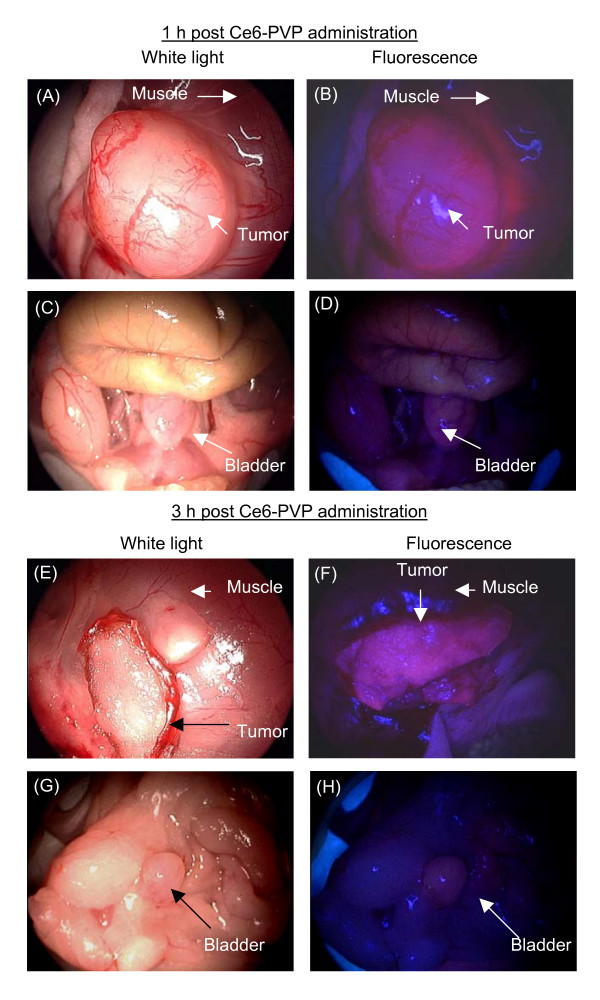
**Macroscopic fluorescence imaging in bladder tumor xenografts and normal bladder at 1 h and 3 h post-intravenous administration of 5 mg/kg Ce6-PVP**. Ce6-PVP induced fluorescence can be characterized by red fluorescence. At both time points, higher fluorescence intensity was observed in the tumor compared to the adjacent muscle and normal bladder of the mice.

**Figure 4:**
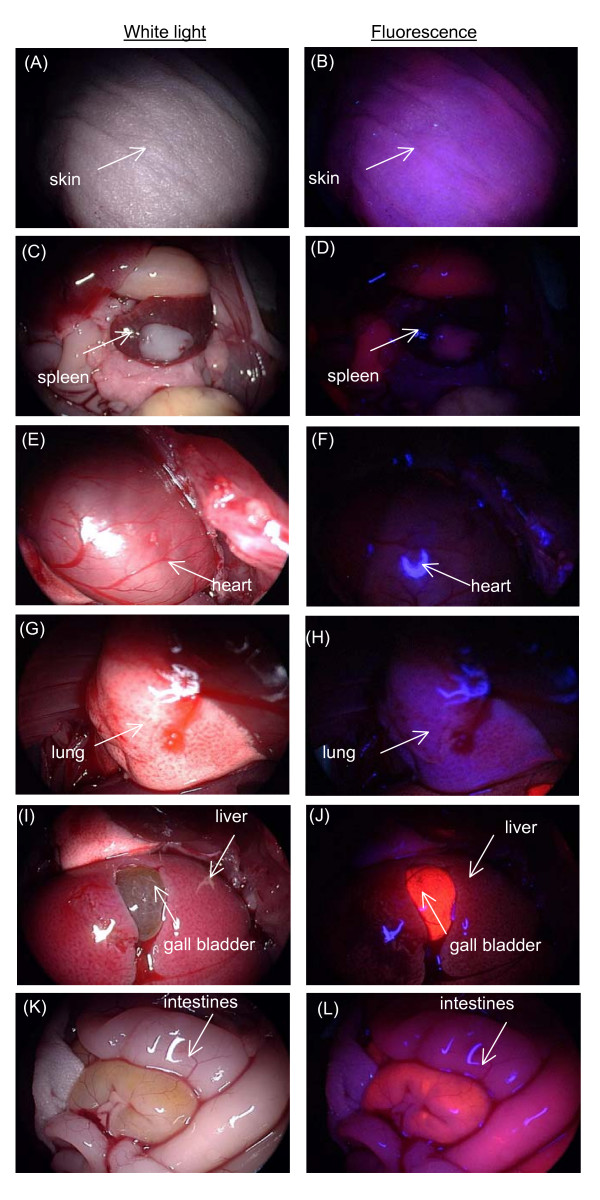
**Macroscopic white light and fluorescence imaging in skin, heart, lung, gall bladder, liver, spleen, kidney and gastrointestinal tract at 1 h post-intravenous administration of 5.0 mg/kg Ce6-PVP**.

**Figure 5:**
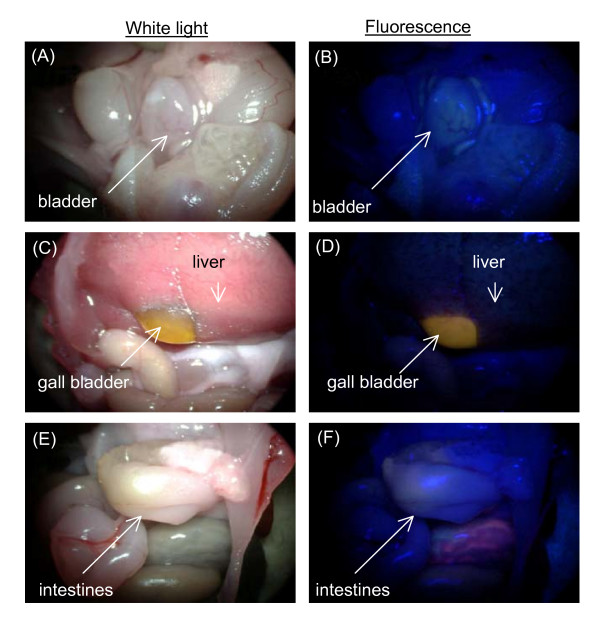
**Macroscopic white light (A, C, E) and fluorescence (B, D, F) imaging in normal bladder, liver, gall bladder, and gastrointestinal tract at 6 h post-intravenous administration of 5.0 mg/kg Ce6-PVP**.

Fluorescence imaging was performed on the bladder tumor xenograft, peritumoral muscle, and normal bladder at 1 and 3 h post intravenous injection of Ce6-PVP in mice (Figure 3). Representative fluorescence images of skin and various internal organs taken at 1 h post Ce6-PVP administration are presented in Figure 4. Overall, tumor fluorescence was observed to be more intense compared to the adjacent peritumoral muscle. Fluorescence intensity in bladder tumor was also higher compare to fluorescence of normal bladder tissues. The internal organs were also found to yield substantial fluorescence especially the gall bladder, liver, stomach, small and gastrointestinal tracts. Minimal fluorescence was observed in the heart and spleen. The fluorescence intensity in muscle, skin, lung, liver, and bladder dropped at 3 h post drug administration. There was little or no fluorescence remaining in heart and spleen. In contrast, the tumors showed sustained fluorescence intensity at 3 h post drug administration. By 6 h post drug administration, minimal fluorescence was detected in the gastrointestinal tract, liver and bladder (Figure 5).

### In vivo fiber optic spectrofluorometric measurement

**Figure 6:**
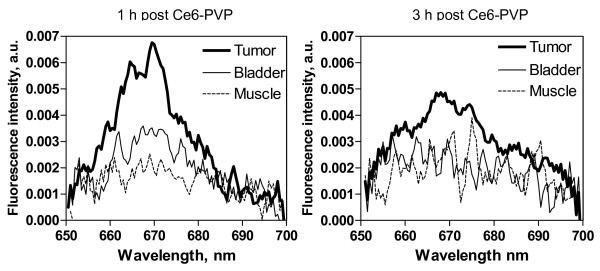
**Comparison of emission spectra of bladder tumor xenograft, normal bladder and muscle of the murine model at 1 and 3 h post administration of Ce6-PVP using 400 nm excitation**. The spectral signatures showed a peak at the wavelength 665 – 670 nm in tumor while the fluorescence intensity of normal bladder and muscle is weaker than that of the tumor tissue.

**Figure 7:**
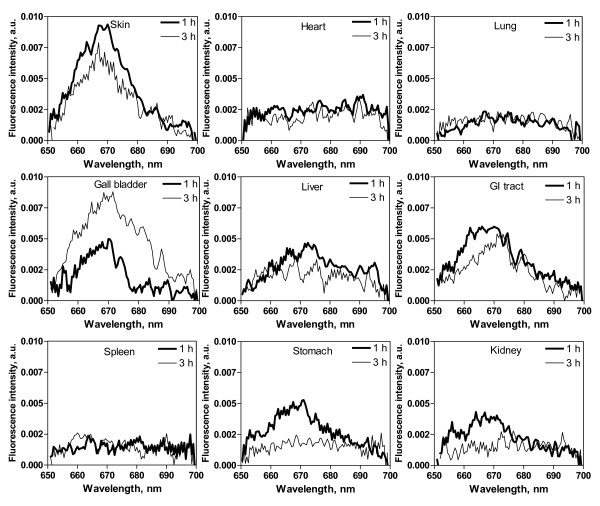
**Comparison of emission spectra in the 650 – 700 nm region using 400 nm excitation in various normal organs at 1 and 3 h post Ce6-PVP administration**. Except for skin and gall bladder, it is evident that the emission spectra of normal organs were lower compared to the emission spectra of tumor.

Typical fluorescence emission spectra from tumor, adjacent peritumoral muscle and normal bladder after i.v. administration of Ce6-PVP are shown in Figure 6. In general, the peak fluorescence intensities of tumor were higher than those of normal sites. The greatest intensity occurred in the region between 660 – 670 nm. When the spectra were normalized to baseline value, changes in peak intensity became evident. The line-shape differences were predominantly due to increased Ce6-PVP accumulation of tumor in the red region (emission peak at 665 nm). Fluorescence emission spectra of skin, heart, lung, gall bladder, liver, spleen, kidney and gastrointestinal tract are shown in Figure 7. Except for the gall bladder, all other organs showed a decreased of fluorescence emission of Ce6-PVP at 3 h compared to 1 h post drug administration. Essentially, fluorescence images that showed greater red fluorescence intensity had visibly higher spectral peak, thus suggesting that that the macroscopic fluorescence imaging were reproducible.

### Fluorescence imaging and spectrofluorometric measurement in a patient

**Figure 8:**
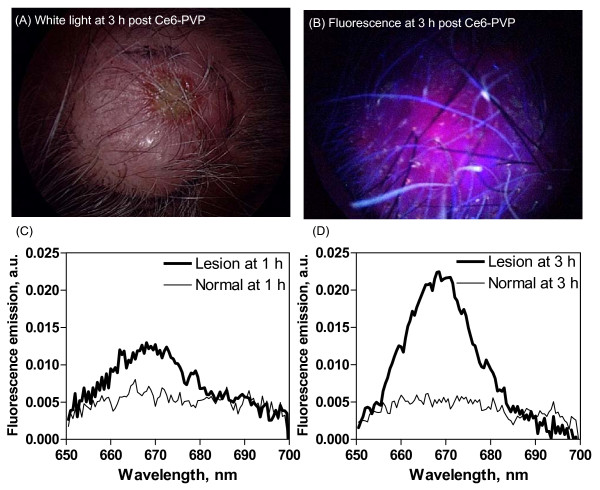
**Fluorescence imaging and spectrofluorometric measurement of fluorescence emission in angiosarcoma lesion and surrounding normal skin on the scalp at 1 h and 3 h post-intravenous administration of 2.0 mg/kg Ce6-PVP**.

Fluorescence imaging and spectroscopy carried out on 3 tumors in an angiosarcoma patient showed that the tumors developed maximum fluorescence emission intensity at 3 h post Ce6-PVP administration (Figure 8). No observable variations were found for the intensity of the fluorescence between tumors. The fluorescence kinetics study findings from this consistent to those of our earlier results [17]. Spectra from tumor areas show a clear distinct Ce6-PVP induced fluorescence spectrum that discriminate between the tumor and normal skin, with tumor showing higher fluorescence emission intensity compared to normal tissue.

## Discussion

Fluorescence imaging approaches are increasingly being used as a medical diagnostic procedure to assess tissue malignancy over conventional methods because they do not use potentially harmful ionizing radiation [20]. In situations where discrimination of suspicious lesion is clinically problematic, fluorescence imaging may provide added advantage in demarcating abnormal tissue. The development of photosensitizer based fluorescence imaging is hindered by problems such as skin photosensitivity, poor selectivity of the photosensitizer, and formulation issues. For these reasons, Ce6 was formulated with PVP to address these issues. Formulations using biocompatible polymers such as PVP are increasingly being used in the pharmaceutical industry for enhancing drug solubility and bioavailability. Complexation of Ce6 with PVP was found to prevent Ce6 aggregation in aqueous media and led to an enhancement of Ce6 fluorescence quantum yield, while keeping the quantum yield of the intersystem crossing essentially unchanged [21]. In this study we have further examined the potential clinical use of Ce6-PVP in cancer imaging and diagnosis. We first tested out the feasibility of this photosensitizer as an exogenous fluorophore on the CAM tumor model. We were able to observe the red fluorescence emitted by the tumor tissue excited using a filtered xenon lamp excitation, which enabled clear determination of the tumor margin. The sensitivity of Ce6-PVP was more than 80%, however, the specificity remained low. We have recently reported that the new formulation of Ce6-PVP (> 95% purity level of active Ce6) demonstrated a higher sensitivity (98%) and specificity (82%) on the CAM model [15].

Results from the CAM experiments have provided the motivation to examine Ce6-PVP fluorescence distribution in bladder tumor xenograft as well as in various normal organs of a murine model. Macroscopic fluorescence imaging showed that there was considerable distinction in the localization of fluorescence in tumor compared to other organs that could enable discrimination between tumor and normal organs. Organs of elimination and detoxification such as skin, gall bladder and gastrointestinal tracts were characterized by high photosensitizer accumulation efficiency. In contrast, all other normal organs such as muscle and bladder had much lower photosensitizer accumulation at 1 h post drug administration. Blood vessels growing on the tumor can also be observed because they contrast with the fluorescence of the tumors. At 3 – 6 h post drug administration, a decrease of fluorescence intensity became evident on all normal organs, confirming that Ce6-PVP has fast clearance rate from normal organs. In some instances, we have observed variability of fluorescence intensity on the surface tissue such as stomach and lung. This is possibly attributed to the variations of the tissue optical properties of the organs given by their color, density and composition.

The key issue in fluorescence imaging is that the emitted fluorescence intensity measured from a tissue surface is not necessarily proportional to the fluorophore concentration because the light is altered by the tissue's intrinsic absorption and scattering properties. Hence we have employed the utility of spectrometric point fluorescence detection as a complementary technique. Spectra measurements were carried out at 1 and 3 h post drug administration to correlate the tumor intensity ratios obtained with fluorescence imaging to the tumor fluorescence spectral signal of the tissue. All the macroscopic fluorescence images correlated well to the spectra measurement. The Ce6-PVP induced spectra emission after normalization demonstrated a good separation to differentiate malignant tumors from normal tissues. Besides measuring physical parameters such as concentration of photosensitizer and tissue properties, this method can potentially improve the assessment of cancer location and its extent within the local-regional area. While fluorescence point spectroscopy studies are promising, it has several drawbacks as a screening tool as it can only interrogate a small volume of tissue (typically, 0.5 – 1 mm^3^) directly beneath the probe tip. Point measurements inevitably involve a degree of random sampling, which may not allow identification of early stage disease [22]. Hence, the combination techniques of fluorescence imaging and spectroscopy have been proven in good agreement with the actual tumor boundary found by histopathological mapping and early stage of disease [23,24].

Recently, we have reported photodynamic therapy – activated immune response against distant untreated tumours in recurrent angiosarcoma [25] and preferential accumulation of Ce6-PVP in angiosarcoma compared to normal skin following intravenous administration in 3 patients [17]. High dose PDT carried out at a high fluence rate resulted in local control of the disease for up to a year; however, the disease recurred and PDT had to be repeated [26]. During the repeat PDT session, we measured the fluorescence of 3 different lesions using fluorescence imaging followed by spectroscopy. The results confirmed that fluorescence imaging clearly captures the fluorescence in angiosarcoma and good correlation was found between fluorescence imaging and spectral measurement in the patient. This is in agreement with other reports, that fluorescence ratio imaging in combination with relative spectral measurement of the photosensitizer might be a viable method for the optical diagnosis of cancer [27]. Furthermore, in vivo and real-time determination of the time course of photosensitizer's fluorescence could potentially be a crucial pre-irradiation screening tool to determine the exact location and extent of the tumor before photodynamic therapy.

## Conclusion

It is shown that Ce6-PVP has a rapid accumulation in the tumor, and a relatively short half-life in normal organs. When excited by blue light, Ce6-PVP accumulating cells can be visualized and located in the tissue by virtue of its fluorescence. The main advantage of Ce6-PVP induced fluorescence imaging is its increased tumor selectivity with the ability to clearly define the tumor margin. Combination of Ce6-PVP induced fluorescence imaging and spectroscopy could allow detection and discrimination between cancer and the surrounding normal tissues.

## Competing interests

The authors declare that they have no competing interests.

## Authors' contributions

WWC and PST conceived of the study and carried out all the experimental study. RB and KCS participated in the clinical study. PWH and MO participated in the coordination of the study. All authors read and approved the final manuscript

## Pre-publication history

The pre-publication history for this paper can be accessed here:

http://www.biomedcentral.com/1471-2342/9/1/prepub
